# Preparation and Application of Magnetic Microporous Organic Networks for Rapid Adsorption Enrichment of Multiple Mycotoxins in Complex Food Matrices

**DOI:** 10.3390/foods14233984

**Published:** 2025-11-21

**Authors:** Chuang Wang, Jing Zhang, Yu-Xin Wang, Dan-Dan Kong, Jian-Xin Lv, Yuan-Yuan Zhang, Xue-Li Li, Xin-Xin Kang, Meng-Yue Guo, Jiao-Yang Luo, Mei-Hua Yang

**Affiliations:** 1 State Key Laboratory for Quality Ensurance and Sustainable Use of Dao-di Herbs, Institute of Medicinal Plant Development, Chinese Academy of Medical Sciences & Peking Union Medical College, Beijing 100193, China; wc19970311@163.com (C.W.);; 2 Key Laboratory of Bioactive Substances and Resources Utilization of Chinese Herbal Medicine, Ministry of Education, Institute of Medicinal Plant Development, Chinese Academy of Medical Sciences & Peking Union Medical College, Beijing 100193, China; 3 Hainan Branch of the Institute of Medicinal Plant Development, Chinese Academy of Medical Sciences & Peking Union Medical College, Haikou 570311, China

**Keywords:** magnetic microporous organic network, mycotoxins, magnetic solid-phase extraction, adsorption mechanism, complex matrix

## Abstract

Mycotoxins commonly contaminate grains and traditional Chinese medicinal materials, posing serious health risks to humans and animals. To address this issue, a magnetic microporous organic network (MMON) was synthesized via an in situ growth method and Sonogashira–Hagihara coupling for the simultaneous adsorption of seven mycotoxins, followed by UPLC-MS/MS detection. The optimized MMON featured a high surface area, uniform micropores, and rapid magnetic separation within 5 s. Structural and compositional analyses confirmed its tailored architecture, while DFT calculations revealed a pore confinement effect, π–π stacking, and hydrophobic interactions as the primary adsorption mechanisms. A magnetic solid-phase extraction (MSPE) method using 8 mg of MMON achieved adsorption equilibrium within 10 s in 5 mL of a 4 mg/L mycotoxin standard solution. The material maintained over 95% efficiency across ten reuse cycles at a low cost. Under optimal conditions, an MSPE-UPLC-MS/MS method with a low detection limit (0.002–0.15 μg/L), wide linear range (0.01–100.0 μg/L), large enrichment factor (20.1–21.9), low adsorbent dosage, and short extraction time was developed. The determination of mycotoxins in complex grain-based foods and herbal products was also realized with recoveries of 81.32% to 116.10%. This work offers a rapid, cost-effective, and high-throughput approach for mycotoxin detection, supporting quality control in food and herbal product safety.

## 1. Introduction

Mycotoxins are toxic secondary metabolites produced by fungi such as Aspergillus, Fusarium, and Penicillium. These compounds commonly contaminate grains, traditional Chinese medicines, and processed foods, posing serious health risks to humans and animals [1,2,3,4]. According to the World Health Organization, exposure to mycotoxins is responsible for over one million cases of liver damage, immune suppression, and cancer globally each year (www.who.int (accessed on 1 October 2025)). Aflatoxin B_1_ (AFB1), classified as a Group 1 carcinogen by the International Agency for Research on Cancer (IARC), is highly toxic, with a median lethal dose (LD50) as low as 0.5 mg/kg in rats (https://publications.iarc.fr/ (accessed on 1 October 2025)). Other commonly encountered mycotoxins, namely ochratoxin A (OTA) and deoxynivalenol (DON), demonstrate nephrotoxic, teratogenic, and gastrointestinal toxicities [5,6,7]. Co-contamination is prevalent, with more than 75% of samples exhibiting the presence of two or more mycotoxins when environmental conditions are conducive [8]. AFB_1_ and OTA are frequently detected together in cereals and nuts, whereas DON and zearalenone (ZEN) commonly co-occur in maize-based products. Combined exposure often intensifies toxicity. AFB_1_ and OTA act synergistically to promote hepatotoxicity and carcinogenesis by amplifying oxidative stress and inhibiting DNA repair, while DON and fumonisins (FBs) disrupt intestinal barrier function [9,10]. Although regulatory standards have been established, traditional methods, including high-performance liquid chromatography–mass spectrometry (HPLC-MS), mainly rely on large-scale equipment and require time-consuming sample preparation procedures. Immunoassay-based detection techniques, including Enzyme-Linked Immunosorbent Assay (ELISA), face interference from complex sample matrices, limited capabilities for simultaneous detection of multiple analytes, and challenges with professional operators, leading to high detection costs [11]. These limitations underscore the critical need for efficient and robust sample pretreatment methods that can reduce matrix complexity and pre-concentrate target analytes, thereby enhancing the overall sensitivity and accuracy of downstream detection.

Magnetic solid-phase extraction (MSPE) has gained prominence in recent years for the detection of environmental pollutants and biological toxins, owing to its rapid separation, ease of operation, and high enrichment efficiency [12,13,14]. The effectiveness of MSPE largely depends on the design of magnetic adsorbents. Core–shell materials, such as Fe_3_O_4_@SiO_2_, exhibit improved selectivity and adsorption capacity through surface functionalization [15]. Recently, a double-macrocycle hierarchical COF (CX4-Tph-COF) integrating calix[4]arene and porphyrin was rapidly constructed, which exhibited synergistic host–guest recognition, an excellent size-sieving effect, and a remarkably high adsorption capacity (298.6 mg/g for ZEN) with ultrafast equilibrium (30 s) for the targeted analysis of multiple mycotoxins [16]. Subsequently, a nickel ferrite magnetic calix[4]arene-derived COF (NiFe_2_O_4_@CX4-COF) was fabricated at room temperature, achieving rapid adsorption equilibrium within 3 min and enabling efficient multi-target extraction of Fusarium mycotoxins in cereals with high sensitivity and minimal matrix effects [17]. A carboxyl-functionalized magnetic amide-linked covalent organic framework (Fe_3_O_4_@COF–COOH) synthesized at room temperature was developed, which efficiently extracted eleven structurally diverse mycotoxins from soybeans via π–π stacking, hydrogen bonding, and electrostatic interactions, with MSPE parameters optimized using Plackett–Burman and Box–Behnken designs, achieving high sensitivity and satisfactory recoveries [18]. An MSPE method employing MIL-101(Cr)@Fe_3_O_4_ nanocomposites as adsorbents was successfully applied for the simultaneous purification and enrichment of nine mycotoxins from maize, wheat, watermelon, and melon [19]. Concurrently, magnetic molecularly imprinted polymers (MMIPs) were developed by integrating commercial molecularly imprinted polymers with Fe_3_O_4_, which achieved the highly selective extraction of four aflatoxins from complex pig feed matrices without the need for intricate synthesis, demonstrating exceptional sensitivity and accuracy [20].

Additionally, Jia et al. developed a metal–organic frameworks@MON (MOFs@MON) composite that utilized the large surface area and tunable pore architecture of MOFs to efficiently extract polycyclic aromatic hydrocarbons [21]. However, MOFs are limited by poor water stability and stringent synthesis requirements, including high temperatures and pressures [22]. Microporous organic networks (MONs), an emerging class of porous materials, present a promising alternative due to their adjustable pore sizes, high surface areas, and abundant functional groups (e.g., amino, carboxyl, alkynyl) [23,24,25,26,27]. However, the main issues with current MON-based adsorbents are cost, large-scale production and regeneration issues, practical application and safety assessment, their inability to be employed in multi-complex matrix materials [28,29], and their limited capacity for multi-target adsorption, since the majority of their applications are confined to one or a small number of analytes [30].

This study presents a magnetic microporous organic network (Fe_3_O_4_@SiO_2_@MON), designed via an in situ growth method. Unlike conventional MON-based adsorbents, which often suffer from limited functionality, slow kinetics, or inadequate specificity for multi-toxin capture, our MMON introduces a fundamental advancement through its integrated design. It features a tripartite core–shell architecture: a superparamagnetic Fe_3_O_4_ core for rapid separation (<5 s), a silica interlayer ensuring chemical stability, and a functionally tailored outer MON shell. The pore size and surface chemistry were precisely engineered to create a synergistic, broad-spectrum affinity for structurally diverse mycotoxins. This design translates into superior performance, achieving rapid equilibrium for seven mycotoxins within 10 s—significantly faster than many reported materials—while utilizing only 8 mg of adsorbent. Combined with high recovery in complex matrices, this work provides a more efficient and practical solution for simultaneous mycotoxin detection compared to existing MONs.

## 2. Materials and Methods

### 2.1. Chemicals and Instruments

Standard solutions of seven mycotoxins were sourced from Qingdao Pribolab Biological Engineering Co., Ltd. (Qingdao, China). Reagents including tetrakis(4-ethynylphenyl)methane (TEPM, ≥98% purity), bis(triphenylphosphine)palladium(II) dichloride ((PPh3)2PdCl2, Pd 15.2%), copper(I) iodide (CuI, ≥99%), 1,4-diiodobenzene (98%), 2,5-dibromohydroquinone (98%), 2,5-dibromobenzene-1,4-diamine (98%), tetraethyl orthosilicate (TEOS, 98%), zirconium(IV) chloride (99.5%), FeCl_3_·6H_2_O (99%), 2-aminoterephthalic acid (98%), and iron oxide nanoparticles (Fe_3_O_4_, 200 nm, 99.5% metals basis) were provided by Shanghai Macklin Biochemical Technology Co., Ltd. (Shanghai, China). Additional Fe_3_O_4_ nanoparticles (20 nm, ≥99% metals basis) were acquired from Beijing Zhongkeleiming Daojin Technology Co., Ltd. (Beijing, China). 2,5-Dibromoterephthalic acid (98%) was obtained from Shanghai Aladdin Biochemical Technology Co., Ltd. (Shanghai, China). HPLC-grade acetonitrile, ethanol, and formic acid were sourced from Thermo Fisher Scientific (Waltham, MA, USA). N,N-dimethylformamide (DMF, 99.5%) was supplied by Shanghai Macklin, while hydrochloric acid, sodium hydroxide, and ammonium hydroxide (NH_3_·H_2_O, 25–28%) were procured from Tianjin Beilian Fine Chemical Development Co., Ltd. (Tianjin, China).

Material characterization was performed using various methods: field-emission scanning electron microscopy (FE-SEM, Hitachi SU8020, Tokyo, Japan) with energy-dispersive X-ray spectroscopy (EDS, HORIBA EX-250, Kyoto, Japan); transmission electron microscopy (TEM, JEM-1200EX, Tokyo, Japan); X-ray diffraction (XRD, Bruker D8 ADVANCE, Karlsruhe, Germany); X-ray photoelectron spectroscopy (XPS, PHI VersaProbe III, Chigasaki, Japan); Fourier-transform infrared spectroscopy (FT-IR, Nicolet iS50, La Jolla, CA, USA); water contact angle measurements (KRÜSS DSA100, Hamburg, Germany); nitrogen adsorption–desorption analysis (ASAP 2460, Lincoln, NE, USA); thermogravimetric analysis (TGA, NETZSCH STA 449, Dresden, Germany); and vibrating sample magnetometry (Lake Shore 8604, Washington, DC, USA). Mycotoxins quantification was conducted via liquid chromatography–tandem mass spectrometry (UPLC-MS/MS, SCIEX Triple Quad 5500, Redwood City, CA, USA), using deionized water (Milli-Q Integral 5, Bay City, MI, USA) to prepare aqueous solutions.

### 2.2. Synthesis Procedures

#### 2.2.1. Synthesis of MMON

**Figure 1:**
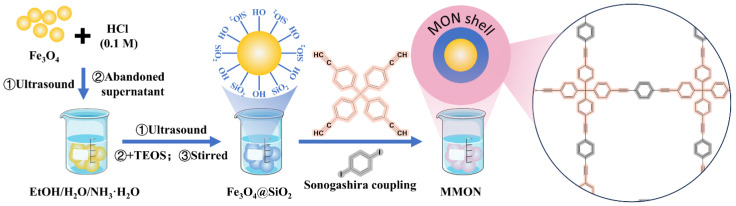
Schematic preparation of MMON.

The synthesis and preparation methods for MMON are illustrated in Figure 1. Fe_3_O_4_@SiO_2_ nanoparticles were synthesized as per the procedure of Wang et al. [31]. Briefly, 1.0 g of Fe_3_O_4_ particles was treated with 200 mL of 0.1 M HCl in a 1 L beaker under ultrasonic conditions for 30 min, followed by three washes with ultrapure water. The magnetic microspheres were then re-dispersed in a solution containing ultrapure water (80 mL), NH_3_·H_2_O (5.0 mL, 25–28%), and ethanol (320 mL). To this, 1.0 mL of TEOS was added, and the system was sonicated for 15 min. The reaction was continued by mechanical stirring for 12 h. The particles were subsequently separated using a magnet, washed with ethanol and ultrapure water, and dried under vacuum.

For the room-temperature synthesis of MMON, Fe_3_O_4_@SiO_2_ NPs (400 mg), CuI (8.8 mg, 0.012 mmol), and (PPh3)2PdCl2 (33.6 mg, 0.012 mmol) were dissolved in a mixture of triethylamine (30 mL) and toluene (30 mL) in a dry flask. After adding TEPM at 200 mg 0.48 mmol) and 1,4-diiodobenzene (317 mg, 0.96 mmol), the mixture was stirred at 400 rpm for 24 h at room temperature to synthesize MMON. The precipitates were then collected by centrifugation (11,291× *g*, 10,000 rpm, 5 min), washed with dichloromethane and absolute ethanol, and dried under vacuum overnight at room temperature (Figure 1).

#### 2.2.2. Synthesis of MOFs

Fe_3_O_4_@SiO_2_ (150 mg), FeCl_3_·6H_2_O (0.6757 g, 2.5 mmol), and 2-aminoterephthalic acid (0.226 g, 1.25 mmol) were dissolved in 15 mL of DMF under ultrasonic treatment to form a homogeneous solution. The solution was then transferred into Teflon-lined autoclaves and heated at 110 °C for 24 h. After cooling to room temperature, the product was isolated using an external magnet, washed five times with DMF and methanol, and dried under vacuum to obtain MIL-101 [21].

UiO-66 was synthesized using a solvothermal method [23]. Fe_3_O_4_@SiO_2_ (150 mg), zirconium (IV) chloride (300 mg, 1287 μmol), and 75 μL of water were dissolved in 20 mL of DMF and stirred for 15 min. 2-aminoterephthalic acid (235 mg, 1298 μmol) in 10 mL of DMF was added to this mixture, followed by stirring until complete dissolution. The mixture was then transferred into Teflon-lined autoclaves and heated at 120 °C for 24 h. Following cooling, the brownish magnetic product was separated, rinsed five times with ultrapure water, and vacuum-dried.

### 2.3. MSPE Procedure and Batch Experiments

Batch experiments were conducted to evaluate the mycotoxin adsorption behavior of various materials. Recovery rates served as the efficiency indicator for the materials.

In each trial, 5 mL of a mycotoxin standard solution (4 mg/L, acetonitrile/water, 1:9) was combined with 5 mg of MMON. The mixture was vortexed for 10 s, then placed in a metal bath shaker at 1000 rpm for a set period, allowing for thorough adsorption. The adsorbent was separated from the solution using an external magnetic field, and the supernatant was discarded. To elute the mycotoxins, 1 mL of elution solution was added and sonicated for a specified time. The adsorbent was separated again. The process was repeated twice for the sufficient elution of the mycotoxins, and the elution solutions were combined. The eluate that had been collected was subjected to nitrogen purging, subsequently re-dissolved to a volume of 0.2 mL, and then quantified using UPLC-MS/MS with a filter of 0.22 μm porosity.

### 2.4. Optimization of MSPE Parameters

Adsorbent dose, adsorption duration, solution pH, desorption solvent, ionic strength, desorption time, and pH were all evaluated using the single-factor optimization approach to see how they affected the material’s adsorption efficiency utilizing the MSPE technique. The rate of mycotoxin recovery was used to evaluate adsorption effectiveness.

### 2.5. Preparation of Standard and Real Samples

First, 1 mg of mycotoxins was dissolved in 1 mL of acetonitrile to create a stock solution of mycotoxins (1 g/L). Before use, the stock was gradually diluted using a 1:9 acetonitrile/water mixture to create the working solution.

Areca nut (Areca catechu), Coix seed (Coix lacryma-jobi), Platycladi seed (Platycladus orientalis), Spine Date seed (Ziziphus jujuba var. spinosa), Barley, and Malt were sourced from a traditional medicine market in Bozhou, Anhui, while Peanut and Corn were purchased from local supermarkets. A methanol/water solvent combination (8:2 ratio) was used for two ultrasonic extractions. After combining the extracts, they were centrifuged for 10 min at 11,291× *g* (equivalent to 10,000 r/min). The final solution was then stored at 4 °C for later use after the supernatant was collected. The optimized MSPE technique was used to evaluate the actual samples. All samples were filtered using a 0.22 μm filter to eliminate any suspended particles before analysis.

### 2.6. Mechanism Analysis

The structures of seven mycotoxins and MON were optimized using the Gaussian method, and their vibrational frequencies were calculated to ensure the absence of imaginary frequencies. Dimer models were constructed based on the optimized structures in Gaussview, with their frequencies calculated to confirm the absence of imaginary frequencies. Weak interaction diagrams for the dimers were generated using Multiwfn and VMD, and the adsorption mechanism was analyzed by correlating the results with XPS data.

DFT calculations were performed using Gaussian 16, Rev. C01. The B3LYP hybrid functional [32], in combination with Grimme’s empirical dispersion correction (D3BJ) [33,34] and the 6-31G(d) basis set [35], was employed for geometry optimization. Harmonic vibrational frequency analysis was conducted at the same level to ensure that the optimized structures exhibited only positive frequencies. NCI calculations and visualizations were executed using Multiwfn 3.7 and VMD 1.9.4 software [36,37].

### 2.7. UPLC-MS/MS Conditions

Quantitative analysis was performed using a SCIEX Triple Quad™ 5500 UPLC-MS/MS system (AB Sciex Pte. Ltd., Singapore). Separation was achieved on a CAPCELL CORE C18 column (2.1 mm I.D. × 50 mm, 2.7 μm, SHISEIDO, Tokyo, Japan), with a mobile phase of 0.1% formic acid in methanol/acetonitrile (1:1, *v*/*v*) and 0.1% formic acid in water. The injection volume was 2 μL, and the flow rate was maintained at 0.3 mL/min. Additional UPLC-MS/MS parameters are detailed in Tables S1 and S2.

### 2.8. Data Analysis and Statistical Methods

Data acquisition and instrumental control were performed using the Analyst Software 1.6.2 on the SCIEX Triple Quad™ 5500 UPLC-MS/MS system. Calibration curves were generated and regression analysis was conducted using Origin graphing and data analysis software. Matrix effects (ME) were assessed by comparing the analyte response in matrix-matched standard solutions prepared using the MSPE procedure with that in pure aqueous standards. The ME was calculated using the formula ME (%) = [(A/B) − 1] × 100, where A is the peak area of the analyte in the matrix-spiked sample extract and B is the peak area of the corresponding pure solvent standard. Results for recovery studies are presented as mean ± standard deviation (*n* = 5). Data obtained from the optimization experiments were expressed as the mean ± standard deviation. All data analysis and statistical comparisons were performed using OriginPro (Version 2025b Learning Edition, OriginLab Corporation, Northampton, MA, USA). To statistically evaluate the significance of the observed differences in mean recovery under various MSPE conditions, one-way analysis of variance was applied. A *p*-value of less than 0.05 was considered statistically significant.

## 3. Results

### 3.1. Synthesis and Characterization of MONs

This study evaluated MMON as an MSPE adsorbent for the simultaneous enrichment and detection of seven mycotoxins. For five different materials (Fe_3_O_4_, Fe_3_O_4_@SiO_2_, MMON, UiO-66, and MIL-101), Fe_3_O_4_ cores of two different sizes (20 nm and 200 nm) were employed as magnetic cores. Fe_3_O_4_ and Fe_3_O_4_@SiO_2_ served as controls to assess the enhancement provided by MMON. The two core sizes enabled investigation of particle size effects on adsorption performance. All composites were systematically characterized to confirm their structural and functional attributes.

**Figure 2:**
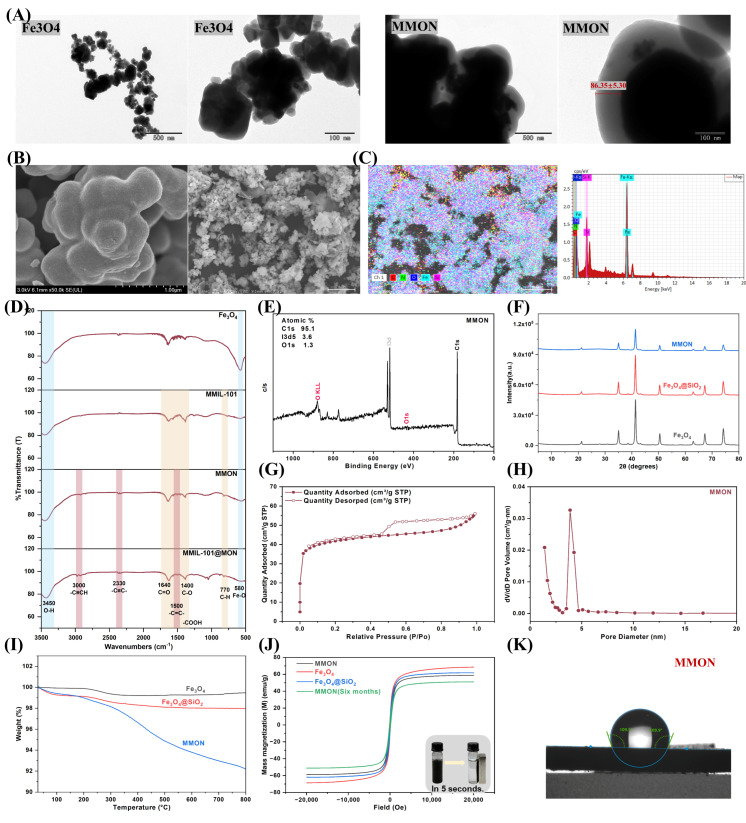
Characterization of adsorptive materials. (**A**) TEM image of Fe_3_O_4_ and MMON. (**B**) SEM image of MMON. (**C**) EDS image of MMON. (**D**) FT-IR spectra. (**E**) XPS spectra of MMON. (**F**) XRD spectra of three materials. (**G**) N2 adsorption–desorption isotherms. (**H**) Pore size distribution of MMON. (**I**) TGA of three materials. (**J**) Magnetic hysteresis curves of different materials. (**K**) Water contact angle analysis of the MMON.

TEM confirmed the successful synthesis of MMON based on its morphology and particle size. The coating thickness, measured using ImageJ 1.53K, was 86.35 ± 5.3 nm (Figure 2A). SEM revealed uniformly spherical particles with a dense microporous surface layer (Figure 2B). EDS analysis (Figure 2C) verified the core–shell architecture, comprising a Fe_3_O_4_ core, a SiO_2_ intermediate layer, and an outer MON. FT-IR spectroscopy (Figure 2D) further confirmed the chemical composition. Characteristic peaks at 580 cm^−1^ (Fe–O) and 3420 cm^−1^ (O–H) indicated the presence of the Fe_3_O_4_ core. Peaks at 1640 cm^−1^ and 1400 cm^−1^ corresponded to C=O and C–O stretching vibrations of carboxyl groups, while a peak at 770 cm^−1^ reflected C–H bending of the benzene ring. Additional peaks at 3000 cm^−1^ (–C≡CH), 2330 cm^−1^ (–C≡C–), and 1500 cm^−1^ (–C=C–) supported the successful formation of the MON shell. XPS (Figure 2E) confirmed the presence of O, C, and I, consistent with the expected MMON composition. The absence of Fe peaks in the XPS spectrum of MMON is the result of the inherent surface analysis characteristics of XPS technology (with a probing depth of only about 10 nm) combined with the core–shell structure encapsulated by multiple layers of thick shells. This is usually proof of a complete encapsulation and a dense shell layer. XRD patterns (Figure 2F) displayed the characteristic face-centered cubic peaks of Fe_3_O_4_ within the 20–70° range, validating the crystalline structure of the magnetic core. The reduced XRD peak intensities of Fe_3_O_4_@SiO_2_@MON are attributed to X-ray absorption and incoherent scattering by the amorphous MON shell, while the unchanged peak positions confirm the retained crystallinity of the Fe_3_O_4_ core. Nitrogen adsorption–desorption analysis (Figure 2G,H) showed that MMON possesses a high specific surface area of 159 m^2^/g, providing abundant active sites for adsorption. Its pore size and volume were 2 nm and 0.09 cm^3^/g, respectively, comparable to those of high-performance adsorbents [28,38], indicating strong adsorption potential. TGA (Figure 2I) revealed minor mass loss near 100 °C due to water evaporation and less than 10% total loss at 800 °C, demonstrating excellent thermal stability. Vibrating sample magnetometry (VSM, Figure 2J) showed a saturation magnetization of 58 emu/g, slightly lower than that of pure Fe_3_O_4_ (68 emu/g) but sufficient for rapid magnetic separation within 5 s. Six-month storage further confirmed the material’s magnetic and structural stability. Water contact angle analysis (Figure 2K) indicated hydrophobicity (109.7 ± 0.2°, >90°).

### 3.2. Adsorption Performance

**Figure 3:**
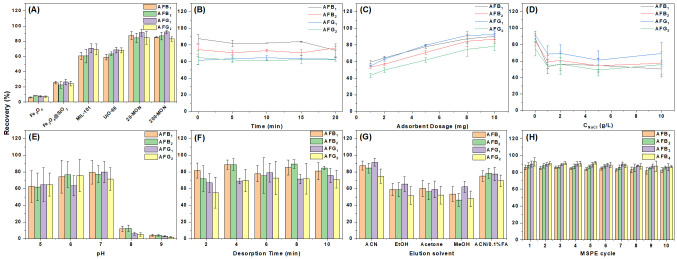
Optimization of MSPE conditions (*n* = 6). (**A**) Evaluation of adsorption for different materials. (**B**) Evaluation of the effect of different adsorption times. (**C**) Evaluation of the effect of different adsorbent usage. (**D**) Evaluation of the effect of ionic strength. (**E**) Impact assessment of pH. (**F**) Evaluation of the effect of desorption time. (**G**) Evaluation of the effect of desorption reagents. (**H**) Evaluation of the reusability.

The adsorption performance of the synthesized materials was assessed through batch experiments. Initial screening using a 10 μg/L mixed (1 mL) (Figure S1) mycotoxin standard showed that several MON-based materials achieved nearly complete adsorption (~100%). However, due to the low concentration and limited differentiation—especially for aflatoxins AFB1, AFB2, AFG1, and AFG2—the standard concentration was increased to 4 mg/L (5 mL). All experiments were conducted using 5 mg of adsorbent (Sartorius balance, ±0.1 mg accuracy). Follow-up tests focused on the most effective candidates. As shown in Figure 3A, at 4 mg/L, distinct differences in adsorption efficiency emerged. Among the tested materials, MMON showed the highest performance, with approximately 80% recovery for the four aflatoxins. Owing to its consistently superior adsorption capacity, MMON was selected for subsequent experiments.

### 3.3. Optimization of MSPE Parameters

MSPE conditions were optimized using 4 mg/L four-aflatoxin standard solution by individually evaluating key parameters, including pH, salt concentration, adsorbent dosage, adsorption time, desorption solvent, and desorption time.

#### 3.3.1. Adsorption Time

Efficient interaction between the adsorbent and sample is crucial for achieving adsorption equilibrium in MSPE. This study evaluated the impact of metal bath oscillation time on adsorption efficiency. As shown in Figure 3B, complete adsorption was achieved with only 10 s of vortexing after mixing the adsorbent and sample—no further agitation was necessary. This rapid adsorption reflects the high availability of active sites and effective mycotoxin capture. Accordingly, a 10 s vortex without metal bath oscillation was determined to be the optimal extraction condition, with 61.5–87.6% recovery for the four aflatoxins, significantly reducing MSPE time.

#### 3.3.2. Adsorbent Dosage

The adsorbent dosage directly affects the number of available binding sites, though excessive amounts may hinder performance. This study assessed dosages of 1, 2, 5, 8, and 10 mg, revealing a steady increase in mycotoxin recovery up to 8 mg, beyond which recovery plateaued (Figure 3C). An 8 mg dose provided sufficient active sites for efficient adsorption, with 74.8–88.7% recovery for the four aflatoxins. Surface functional groups on the MMON adsorbent facilitated strong mycotoxin binding via hydrophobic and π–π interactions, promoting rapid and effective enrichment. To optimize both efficiency and resource use, 8 mg was selected for further experiments.

#### 3.3.3. Ionic Strength

The impact of ionic strength on MMON-based MSPE was assessed using 10 g/L NaCl. As shown in Figure 3D, extraction efficiency for all four mycotoxins declined but remained near 60%, indicating resistance to ionic interference. This stability supports its suitability for complex sample matrices.

#### 3.3.4. pH

Sample solution pH affects MSPE efficiency by modifying the ionization states of functional groups on both mycotoxins and the MMON adsorbent. The pH was adjusted using 0.1 M HCl or NaOH. As shown in Figure 3E, adsorption was most effective between pH 5.0 and 7.0 (61.9–64.8% recovery for pH 5.0, 63.5–76.7% recovery for pH 6.0, and 71.5–79.8% recovery for pH 7.0). When the pH exceeded 7.0, a decrease in adsorption efficiency was observed, which may be attributed to the reduced stability of certain mycotoxins, such as aflatoxins, under alkaline conditions (3.8–11.7% recovery for pH 8.0, and 2.0–4.2% recovery for pH 9.0). Therefore, pH 7.0 was selected for subsequent experiments to ensure both optimal adsorption and analyte stability.

#### 3.3.5. Desorption Conditions

The impact of desorption on detection efficiency is critical. Thus, this study evaluated the effects of desorption time and solvent choice on desorption efficiency. Acetonitrile was initially selected as the eluent. As shown in Figure 3F, complete desorption of mycotoxins was achieved after 4 min of ultrasonication, with 69.7-88.8% recovery for the four aflatoxins. A comparative evaluation of various solvents (Figure 3G) confirmed acetonitrile as the most effective under the tested conditions, with 74.8-91.5% recovery for the four aflatoxins. These results demonstrate that employing MMON in MSPE for mycotoxin enrichment not only significantly reduces extraction time, sorbent amount, and reagent consumption, but also offers additional benefits, including pH stabilization and resistance to ionic interference—both crucial for accurate mycotoxin quantification in complex matrices.

The optimized MSPE procedure was as follows: 8 mg of MMON was mixed with 5 mL of standard or sample solution and vortexed for 10 s to adsorb mycotoxins. The sorbent was then magnetically separated, and the supernatant discarded. Subsequently, 0.5 mL of eluent was added to the sorbent, followed by 4 min of ultrasonication. The sorbent was again separated and the supernatant collected. This elution step was repeated twice, and the collected eluent was nitrogen blow-dried and then reconstituted to a 0.2 mL volume before UPLC-MS/MS quantitative analysis using a filter with a pore size of 0.22 μm.

### 3.4. Method Validation

The calibration curves, accuracy, LOD, linear range (Figure S2), LOQ, and enrichment factor (EF) of mycotoxins were obtained using the developed MMON sample pretreatment and UPLC-MS/MS determination method under optimal MSPE conditions (Table 1). The standard chromatogram of mycotoxins is shown in Figure S3. Each analyte showed strong linearity within its validated range (see Table 1), with correlation coefficients (R2) above 0.995. The LOD and LOQ, defined by signal-to-noise ratios of 3 and 10, respectively, ranged from 0.002 to 0.05 μg/L for LOD and 0.007 to 0.15 μg/L for LOQ. A comprehensive uncertainty estimation and reproducibility assessment was conducted. The intra-day and inter-day precision, expressed as the relative standard deviation (RSD), were determined by analyzing five replicates at the lower end of the linear range. The results confirm the outstanding reproducibility of the method, with intra-day RSDs ranging from 1.9% to 5.6% and inter-day RSDs from 2.1% to 5.8% across all seven mycotoxins. These low RSD values, all well below 6%, provide strong quantitative evidence for the high repeatability and intermediate precision of our method in complex matrices. The consistency between intra-day and inter-day RSDs further attests to the robustness of the analytical procedure.

### 3.5. Reusability and Stability

To reduce costs and improve environmental sustainability, adsorbent recycling was employed. After the initial MSPE, MMON was regenerated by sequentially rinsing with 2 mL of acetonitrile over multiple cycles. As shown in Figure 3H, the mycotoxin peak areas declined by only ~5% after 10 cycles, confirming the sorbent’s durability and reusability. Overall, the MMON-based method delivers excellent linearity, high sensitivity, good precision, and effective reusability for mycotoxin analysis.

### 3.6. Real Sample Analysis

The practical applicability of the MMON-based MSPE method was evaluated using eight real samples, comprising common grains and traditional Chinese medicinal herbs. This selection ensured broad relevance across dietary and medicinal products. The quantitative results are presented in Table 2. The analysis revealed varied mycotoxin contamination profiles: AFB_1_ was detected in Areca nut (0.41 ± 0.13 μg/kg), Coix seed (0.40 ± 0.15 μg/kg), and Peanut (0.54 ± 0.21 μg/kg); AFB_2_ in Platycladi seed (0.37 ± 0.14 μg/kg) and Barley (0.04 ± 0.03 μg/kg); co-contamination of AFG2 and OTA in Areca nut (0.28 ± 0.07 μg/kg and 0.12 ± 0.04 μg/kg, respectively); and ZEN in Corn (0.87 ± 0.27 μg/kg). Method reliability was further validated through recovery studies. Samples were spiked with mycotoxins at 0.5, 5, and 50 μg/L, yielding recovery rates of 81.32–116.10% (Table 3), in line with established criteria.

Matrix effects (ME) were assessed by comparing MSPE with and without MMON. Analyte responses were measured in aqueous and matrix-matched solutions at 50 μg/L, with ME calculated as ME = [(A/B) − 1] × 100%, where A is the peak area of the matrix-spiked sample and B is the corresponding standard. As shown in Table S3, the use of MMON as a sorbent effectively minimized ME. Without MMON, the ME for the target mycotoxins in real samples ranged from −41.99% to 12.37%, indicating severe signal suppression and enhancement. In contrast, the ME was significantly suppressed to a range of −16.91% to 5.24% after the MMON-based cleanup, demonstrating its effectiveness in providing a cleaner extract and mitigating analytical interference. It should be noted that the present study, analyzing eight samples across different matrices, serves as a pilot-scale demonstration. While the results are promising, the sample size is insufficient to broadly generalize the findings on matrix effect suppression. Future work involving a larger and more statistically representative sample set is warranted to confirm the method’s robustness across diverse commodities.

### 3.7. Comparison with Other Methods

The performance of the MMON-based MSPE method was evaluated through comparative analysis (Table 4 and Table S4 [23,39,40,41,42,43,44,45,46]), revealing several distinct advantages. MMON synthesis is simple, low-cost, and energy-efficient, requiring no high temperatures or pressures, with material costs under USD 1 per 8 mg. The economic feasibility of MMON was evaluated through comparative synthesis studies. Its one-pot preparation (yield: greater than 80%) reduces the manufacturing complexity compared to MOFs that require multistep coordination (yield: 40–65%). Replacing expensive metal ligands with Fe_3_O_4_@SiO_2_ results in lower raw material costs, making it cost-effective for high-value food safety applications. The sorbent also exhibits excellent reusability. The method is rapid and environmentally friendly, achieving efficient adsorption in just 10 s with only 8 mg of sorbent [41,42]. Its high-throughput capacity further enhances its applicability [44]. Notably, the method provides a wide linear range and low detection limits for multiple mycotoxins and has been successfully applied to complex food and medicinal matrices [23,39,43]. Collectively, these features highlight the MMON-based MSPE method as a practical, sensitive, and sustainable solution for mycotoxin detection.

### 3.8. Adsorption Mechanism Evaluation

This paper provides an in-depth analysis of the adsorption mechanisms involving π–π interactions and hydrophobic forces. The study integrates DFT simulations, XPS analysis before and after mycotoxins adsorption, and selectivity comparisons to reveal the underlying processes.

The microporous structure enables the entry of analytes smaller than 2 nm, increasing available adsorption sites. Meanwhile, the mesoporous structure of MMON provides size-exclusion properties, preventing non-specific adsorption of larger molecules, such as proteins and enzymes. The 2 nm pore size, 2–3 times the size of mycotoxin molecules, and the tailored pore distribution are key to the high adsorption efficiency observed. Additionally, the large surface area enhances interactions between MMON and target analytes, improving both adsorption capacity and extraction efficiency.

**Figure 4:**
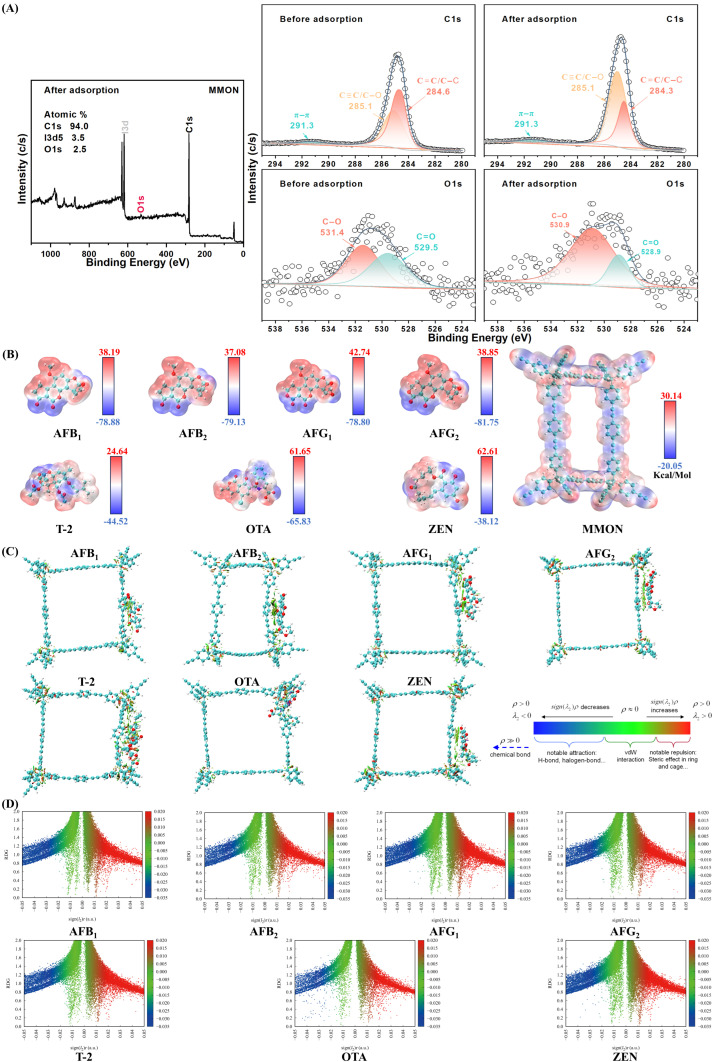
Adsorption mechanism characterization. (**A**) XPS analysis diagram of MMON before and after mycotoxin adsorption. (**B**) Diagram of electrostatic potential for mycotoxins and MMON. (**C**) Diagram of noncovalent interaction analysis of mycotoxins with MMON. (**D**) Scatter plot of noncovalent interactions of mycotoxins with MMON.

XPS analysis was employed to examine the elemental composition of MMON before and after mycotoxin adsorption (Figure 4A). Theoretical simulations further support the role of the pore confinement effect, π–π stacking, and hydrophobic interactions. In the photoelectron spectra (Figure 4A), the change in the survey scan spectrum is mainly reflected in the increase in oxygen atom percentage (before 1.3%, after 2.5%), which provides supporting evidence for the adsorption of mycotoxins by MMON. The C spectrum shows three peaks at 291.3 eV, 285.1 eV, and 284.6 eV, corresponding to C=C bonds on the benzene ring, C≡C/C–O, and C–C/C–H [38,52]. The O spectrum reveals peaks for C–O (531.4 eV) and C–O (529.5 eV) [53]. After mycotoxin adsorption, the binding energies of C=C decrease and the C–O peak area ratio increase, likely due to enhanced shielding from π electron delocalization and the accumulation of hydrophobic groups on the surface. These findings confirm the involvement of π–π stacking, hydrophobic interactions, and electrostatic adsorption between MMON and mycotoxins [28].

Using DFT visualization, electrostatic potential (ESP) maps of seven mycotoxin molecules interacting with MMON were generated (Figure 4B) to assess electrostatic complementarities and identify regions of charge transfer or dipole interactions. ESP maps reveal a pronounced electrostatic complementarity: MMON possesses strongly electron-rich alkynyl triple bonds and aromatic rings (ESP as low as −20.05 kcal/mol) alongside electron-deficient aromatic hydrogens (ESP up to +30.14 kcal/mol). These regions interact complementarily with specific sites on the mycotoxins; for instance, the electron-deficient furan ring double bond of AFB1 (ESP up to +38.19 kcal/mol) aligns with MMON’s electron-rich alkynyl groups, driving adsorption via strong π–π stacking and electrostatic attraction, which is consistent with the decreased C=C binding energy in XPS. Concurrently, the electron-rich lactone carbonyl oxygen of AFB1 (ESP as low as −78.88 kcal/mol) interacts with MMON’s electron-deficient aromatic hydrogens, facilitating dipole–dipole interactions and explaining the increased C–O signal. Noncovalent interaction analyses of seven mycotoxins with MMON (Figure 4C) and scatter plots (Figure 4D) further elucidate the adsorption mechanisms, highlighting prominent π–π stacking interactions. The X-axis reads “Reduced Density Gradient (RDG, Å^−4^)”, quantifying the spatial extent of interactions, while the Y-axis specifies “sign(λ_2_)ρ (a.u.)”, denoting interaction strength and type (negative/positive values indicate attractive/repulsive forces). This labeling directly links to the NCI diagram (Figure 4C), where sign(λ_2_)ρ < −0.03 corresponds to hydrogen bonds (blue isosurfaces in Figure 4C); −0.01 < sign(λ_2_)ρ < 0 reflects van der Waals zones (green isosurfaces); and sign(λ_2_)ρ > 0.02 indicates steric clashes (red isosurfaces). The scatter plot (Figure 4D) statistically validates the spatial interactions depicted in the NCI diagram (Figure 4C). AFB1 points concentrate at sign(λ_2_)ρ = −0.015 ± 0.003 (region II), reflecting van der Waals-driven adsorption in hydrophobic pockets—consistent with green isosurfaces in Figure 4C. Solvation effect analysis (Table S5) reveals that although water solvation weakens π–π stacking due to competitive hydration and dielectric screening, the hydrophobic micropores of MMON create a low-dielectric environment, effectively excluding bulk water. This preserves a significant portion of the π–π binding energy, compensating for solvation losses. In summary, DFT and XPS data reveal two primary interactions between MMON and mycotoxins: (i) mycotoxins preferentially align their benzene rings parallel to the MMON surface, forming an offset face-to-face (OFF) π–π stacking arrangement, and (ii) hydrophobic interactions occur between MMON and mycotoxins.

## 4. Conclusions

This study demonstrates the successful development of MMON via a layer-by-layer assembly, incorporating a Fe_3_O_4_ core, silica interlayer, and functionalized shell. The material exhibits exceptional structural properties, including a high surface area, uniform micropores, and strong magnetization, enabling rapid magnetic separation (<5 s). These features, alongside functionalization, allow MMON to efficiently adsorb seven mycotoxins, as confirmed by TEM, XPS, and FTIR analysis. The optimized MSPE method achieves ultra-fast equilibrium (10 s) with just 8 mg of MMON at pH 7, delivering 80% recovery for 4 mg/L aflatoxins (99% for 10 µg/L) and a detection limit of 0.002 μg/L (AFB_2_). MMON maintains 60% recovery under high ionic strength (10 g/L NaCl) and exhibits >95% reusability after ten cycles, with a cost of less than USD 1 per test (Table S6). DFT simulations and XPS analysis reveal two primary adsorption mechanisms—π–π stacking and hydrophobic interactions—providing a theoretical basis for targeted adsorbent design. In complex real matrices (Areca nut, Coix seed, Platycladi Seed, Spine Date Seed, Barley, Malt, Peanut, and Corn), MMON achieves recovery between 81.32% and 116.10% (RSD < 20%), meeting international standards without complex pretreatment.

Compared to conventional methods, the MMON-based MSPE approach offers high-throughput analysis (simultaneous detection of seven mycotoxins), rapid processing (<15 min, Table S7), and environmental sustainability (minimal adsorbent usage). This method provides a transformative solution for mycotoxin monitoring in food safety and Chinese medicinal herb quality control. The study advances multifunctional adsorbent engineering and underscores the potential of DFT-guided material design for complex matrix applications.

## Figures and Tables

**Table 1:** Validation data of the developed method.

Analyte	Linear Range (µg/L)	Correlation Coefficient (R2)	Linear Fit Equation	LOD (µg/L)	LOQ (µg/L)	EFs	RSD(%) (*n* = 5)	
							Intraday	Interday
AFB1	0.05–20	0.9989	y = −1100.5 + 297596.9x	0.005	0.015	21.3	3.0	3.3
AFB2	0.01–20	0.9983	y = 142.3 + 216831.1x	0.002	0.007	20.8	4.5	4.2
AFG1	0.02–50	0.9985	y = −591.0 + 146631.9x	0.005	0.016	21.8	3.9	3.6
AFG2	0.05–50	0.9981	y = 958.1 + 80828.9x	0.010	0.031	20.9	3.3	3.7
T-2	0.10–50	0.9956	y = 188.8 + 221829.9x	0.020	0.060	21.6	1.9	2.6
OTA	0.20–100	0.9996	y = 3428.8 + 9770.5x	0.050	0.150	21.7	5.6	5.8
ZEN	0.05–100	0.9969	y = −231.5 + 62135.4x	0.010	0.030	20.1	2.3	2.1

**Table 2:** The determination of mycotoxins in real samples (*n* = 5).

Mycotoxins	Found (μg/kg) ± SD							
	Arecae Nut	Coix Seed	Platycladi Seed	Spine Date Seed	Barley	Malt	Peanut	Corn
AFB1	0.41 ± 0.13	0.40 ± 0.15	ND	ND	ND	ND	0.54 ± 0.21	ND
AFB2	ND	ND	0.37 ± 0.14	ND	0.04 ± 0.03	ND	ND	ND
AFG1	ND	ND	ND	ND	ND	ND	ND	ND
AFG2	0.28 ± 0.07	ND	ND	ND	ND	ND	ND	ND
T-2	ND	ND	ND	ND	ND	ND	ND	ND
OTA	0.12 ± 0.04	ND	ND	ND	ND	ND	ND	ND
ZEN	ND	ND	ND	ND	ND	ND	ND	0.87 ± 0.27

Note: SD: standard deviation. ND: not detected.

**Table 3:** Recovery for the determination of mycotoxins in real samples (*n* = 5).

Mycotoxins	Spiked (μg/L)	Recovery ± SD (%)							
		Areca Nut	Coix Seed	Platycladi Seed	Spine Date Seed	Barley	Malt	Peanut	Corn
AFB1	0.5	106.33 ± 4.55	105.59 ± 8.16	90.89 ± 2.56	81.32 ± 6.49	82.55 ± 3.37	82.53 ± 6.56	85.01 ± 7.82	88.17 ± 8.7
	5	102.07 ± 0.80	100.72 ± 2.79	95.42 ± 2.20	91.61 ± 3.36	91.59 ± 8.28	85.36 ± 5.43	93.16 ± 9.98	86.31 ± 4.39
	50	87.15 ± 1.62	89.69 ± 1.68	98.19 ± 2.14	94.52 ± 2.06	92.07 ± 4.28	90.88 ± 1.72	93.44 ± 3.25	92.48 ± 13.78
AFB2	0.5	105.30 ± 5.13	105.59 ± 8.16	87.53 ± 3.93	81.07 ± 2.07	92.94 ± 1.73	91.42 ± 1.57	94.61 ± 1.48	86.25 ± 6.61
	5	98.07 ± 0.95	96.82 ± 1.76	96.52 ± 2.11	90.04 ± 2.07	91.48 ± 2.35	83.66 ± 5.22	89.69 ± 9.96	83.72 ± 4.45
	50	86.53 ± 2.57	85.57 ± 1.73	105.24 ± 3.37	96.25 ± 10.45	99.91 ± 4.61	90.74 ± 1.91	90.56 ± 10.02	98.4 ± 11.35
AFG1	0.5	108.58 ± 6.77	104.29 ± 2.69	87.89 ± 3.65	81.15 ± 7.12	89.75 ± 9.37	90.3 ± 11.53	91.79 ± 12.63	94.73 ± 10.13
	5	103.01 ± 3.49	102.45 ± 2.05	101.38 ± 2.42	99.92 ± 1.62	88.96 ± 1.47	91.43 ± 4.34	86.76 ± 6.62	81.77 ± 3.35
	50	90.63 ± 2.55	90.07 ± 2.24	97.30 ± 2.69	101.52 ± 3.19	89.03 ± 4.24	88.35 ± 1.59	87.18 ± 7.72	94.98 ± 10.11
AFG2	0.5	92.57 ± 5.74	84.72 ± 8.05	85.08 ± 4.64	86.81 ± 10.98	90.57 ± 11.87	92.92 ± 8.89	88.65 ± 12.52	91.22 ± 9.78
	5	97.11 ± 2.73	92.22 ± 4.02	95.13 ± 3.30	96.5 ± 2.32	88.41 ± 9.83	90.18 ± 3.33	84.76 ± 1.18	90.53 ± 3.55
	50	96.16 ± 3.02	94.21 ± 1.43	94.44 ± 3.27	98.15 ± 2.7	88.71 ± 3.22	87.13 ± 1.98	95.46 ± 8.89	83.09 ± 9.39
T-2	0.5	87.04 ± 11.96	99.66 ± 9.53	86.35 ± 2.71	95.32 ± 3.34	84.34 ± 2.43	93.99 ± 7.76	96.4 ± 4.91	86.7 ± 7.31
	5	96.01 ± 6.33	111.81 ± 4.01	86.74 ± 2.65	96.47 ± 1.12	93.95 ± 10.72	87.49 ± 2.12	88.06 ± 1.19	96.2 ± 3.51
	50	100.69 ± 1.45	97.06 ± 2.17	91.24 ± 5.20	94.27 ± 6.35	94.56 ± 1.98	94.66 ± 6.34	98.54 ± 2.21	86.37 ± 6.01
OTA	0.5	89.74 ± 12.58	96.55 ± 18.38	87.76 ± 7.63	89.17 ± 8.83	86.12 ± 4.13	84.08 ± 4.07	86.4 ± 4.80	93.85 ± 6.13
	5	103.86 ± 9.06	105.94 ± 1.88	90.17 ± 1.35	86.63 ± 3.99	87.78 ± 5.22	91.75 ± 2.79	90.56 ± 1.41	89.42 ± 2.34
	50	87.72 ± 4.90	88.15 ± 3.30	103.66 ± 2.86	101.94 ± 10.03	95.83 ± 2.13	86.02 ± 4.30	96.39 ± 2.74	88.25 ± 4.5
ZEN	0.5	99.25 ± 18.19	116.10 ± 15.58	93.97 ± 4.57	93.42 ± 5.77	105.1 ± 12.21	92.99 ± 10.32	91.78 ± 4.08	91.42 ± 12.93
	5	93.24 ± 5.70	111.62 ± 6.01	107.32 ± 4.06	98.61 ± 3.49	89.94 ± 10.01	84.76 ± 6.37	91.22 ± 9.39	96.02 ± 6.11
	50	103.17 ± 4.15	103.57 ± 4.35	91.63 ± 3.67	94.77 ± 9.01	89.90 ± 3.56	98.94 ± 1.18	88.65 ± 7.78	87.21 ± 11.05

Note: SD: standard deviation. The Chinese Pharmacopoeia (2020, Part I) stipulates uniform limits for the detection of aflatoxins in different varieties: no more than 5 μg of AFB1 per 1000 g of Chinese medicinal slices, and the total amount of AFG2, AFG1, AFB2, and AFB1 shall not exceed 10 μg. Only the variety of Coix lacryma-jobi is recorded for the detection of zearalenone, with its limit being no more than 500 μg of zearalenone per 1000 g.

**Table 4:** Comparison of recent LC-MS/MS methods for mycotoxin analysis.

Adsorbent	Method	Matrix	Analytes	Linear Range (µg/L)	Recovery (%)	LOD (µg/L)	Equilibrium Time(s)	Reusability (Cycle)	Ref.
Fe_3_O_4_@COF	MSPE-UHPLC-MS/MS	tomato, strawberry, watermelon, melon, hawthorn	AFB_1_, AFB_2_, AFG_1_, AFG_2_, OTA, OTB, ZEN	0.05–200	74.25–111.75	0.01–0.5	480	1	[47]
MIL-101(Cr)@Fe_3_O_4_	MSPE-UHPLC-MS/MS	Maize, wheat, watermelon, melon	AFB_1_, AFB_2_, AFG_1_, AFG_2_, OTA, OTB, T-2, HT-2, DAS	0.2–100	83.50–108.50	0.02–0.06	240	Not mentioned	[19]
Fe_3_O_4_@PDA/MIL-101(Cr)	MSPE-UHPLC-MS/MS	Licorice	AFB_1_, AFG_1_, STE, ZEN, OTA	0.5–50	78.53–116.28	0.01–0.09	1800	5	[48]
Fe_3_O_4_@PPy	DMSPE- UHPLC-HRMS	Paprika	AFG_1_, AFG_2_, AFB_1_, AFB_2_	3.5–50	81.90–99.40	1.0–1.4	1800	5	[49]
mMIP	SPE-HPLC-MS/MS	wheat, maize	ZEN	5–300	76.00–98.00	0.044	900	Not mentioned	[50]
Fe_3_O_4_@BB-COF	MSPE-UHPLC-MS/MS	soybean, rice, Corn, brown rice and buckwheat	AFB_1_, AFB_2_, AFG_1_, AFG_2_, AFM_1_	0.05–20	76.80–97.10	0.01–0.45	60	6	[51]
MMON	MSPE-UPLC-MS/MS	Arecae nut, Coix seed, Platycladi Seed, Spine Date Seed, Barley, Malt, Peanut, Corn	AFB1 AFB2 AFG1 AFG2 T-2 ZEN OTA	0.01–100	81.32–116.10	0.002–0.15	10	10	This work

## Data Availability

The original contributions presented in the study are included in the article and Supplementary Materials; further inquiries can be directed to the corresponding authors.

## Supplementary Materials

The following supporting information can be downloaded at https://www.mdpi.com/article/10.3390/foods14233984/s1: Figure S1: Comparison of mass spectrometry intensity columns of supernatants after adsorption of 12 materials on a 10 ppb mixed standard of mycotoxins (*n* = 6); Figure S2: Calibration curves of the 7 mycotoxins; Figure S3: Typical extraction total ion current diagram of 7 mycotoxins obtained through the developed method: the concentration is 100 μg/L; Table S1: LC parameters; Table S2: MS/MS parameters; Table S3: Matrix effects of mycotoxins in real sample; Table S4: Comparison of the proposed method with other analytical methods; Table S5: DFT-based analysis of solvation effects; Table S6: MMON synthesis cost and estimated yield per step; Table S7: MSPE procedure time table.
