# Supplementary material for: Preparation and Application of Magnetic Microporous Organic Networks for Rapid Adsorption Enrichment of Multiple Mycotoxins in Complex Food Matrices
Source: Foods. 2025 Nov 21;14(23):3984. doi: 10.3390/foods14233984 (PMC12692691; doi:10.3390/foods14233984)
Supplement: Supplementary file 1 [file foods-14-03984-s001.zip › foods-3978611-supplementary.pdf]

Preparation and Application of Magnetic Microporous Organic Networks for Rapid Adsorption Enrichment of Multiple Mycotoxins in Complex Food Matrices

Chuang Wang[a], DanDan Kong[a], Jing Zhang[a], YuXin Wang[a], JianXin Lv[a], YuanYuan Zhang[a], XueLi Li[a], XinXin Kang[a], MengYue Guo[a], JiaoYang Luo[a,b] *, MeiHua Yang[a,b,c] *

[a]*State Key Laboratory for Quality Ensurance and Sustainable Use of Dao-di Herbs, Institute of Medicinal Plant Development, Chinese Academy of Medical Sciences & Peking Union Medical College, Beijing 100193, China;*

[b]*Key Laboratory of Bioactive Substances and Resources Utilization of Chinese Herbal Medicine, Ministry of Education, Institute of Medicinal Plant Development Chinese Academy of Medical Sciences & Peking Union Medical College, Beijing, 100193, China;*

[c]*Hainan Branch of the Institute of Medicinal Plant Development, Chinese Academy of Medical Sciences & Peking Union Medical College, Haikou 570311, China*

* Correspondence: jyluo@implad.ac.cn (J-Y.L.);yangmeihua15@hotmail.com (M-H.Y.);

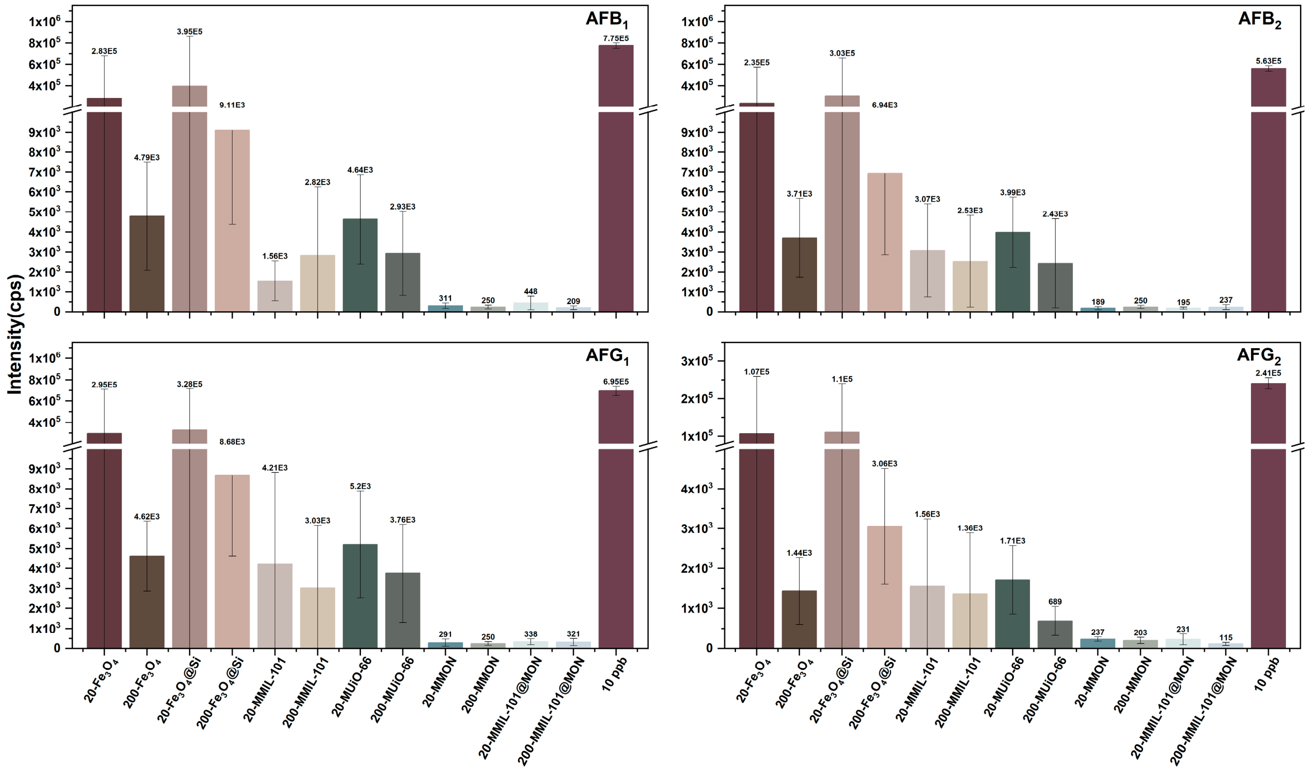

Figure S1 Comparison of mass spectrometry intensity columns of supernatants after adsorption of 12 materials on a 10ppb mixed standard of mycotoxins (n=6).

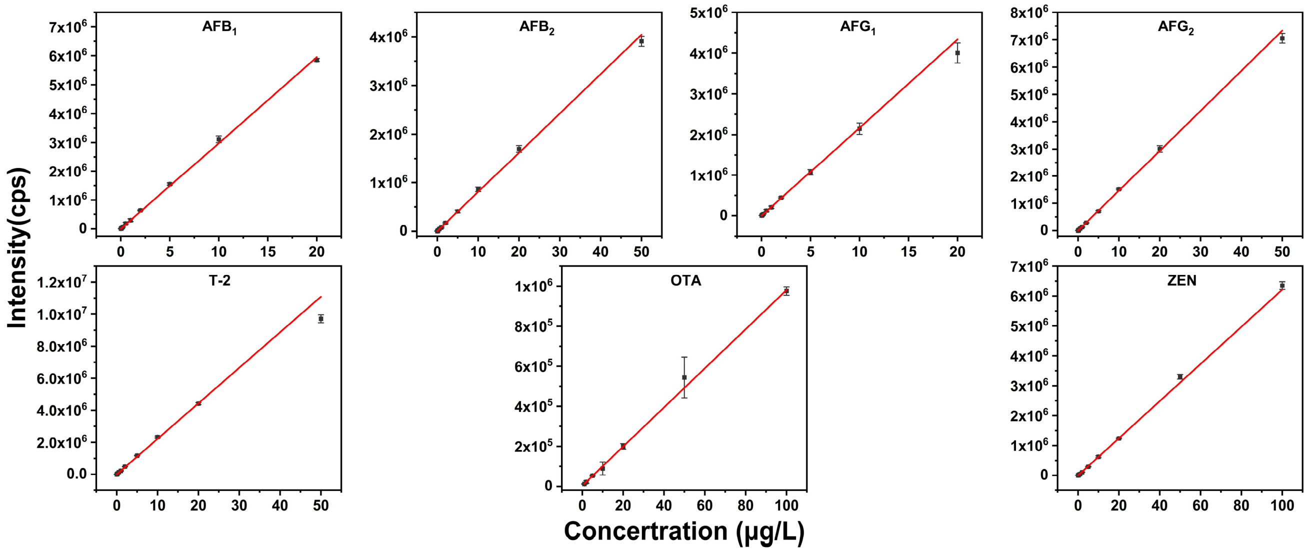

Figure S2 Calibration curves of the 7 mycotoxins.

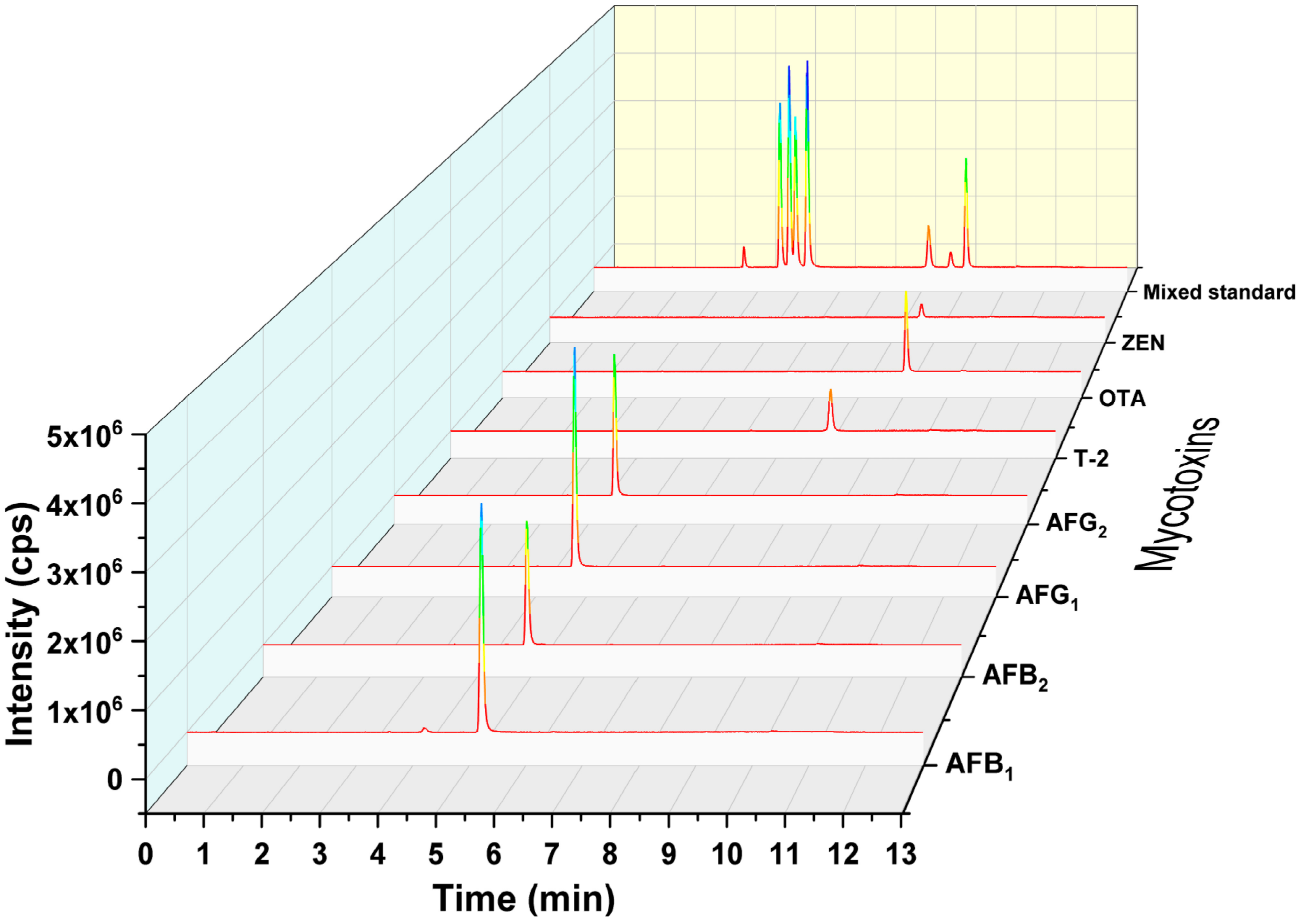

Figure S3 Typical extraction total ion current diagram of seven mycotoxins obtained through the developed method: the concentration is 100 μg/L.

Table S1 LC parameters.

Time(min)	A (%)	B (%)
0~2	5	95
2~2.1	5~40	95~60
2.1~7	40~55	60~45
7~10	55~90	45~10
10~10.5	90~5	10~95
10.5~13	5	95

Separation was performed on a CAPCELL CORE C18 column (2.1 mm I.D. × 50 mm, 2.7 μm, SHISEIDO, Japan). The A phase was 0.1% formic acid–methanol/acetonitrile (1:1), and the B phase was 0.1% formic acid/water. The injection volume was 2 μL and the flow rate was 0.3 mL/min.

Table S2 MS/MS parameters.

Mycotoxin	Mass weight	Mode	Precursor ion (m/z)	Product ion (m/z)	CE (V)
AFB_1	312.1	ESI^+	313.1	285.1	32
				241.1	50
AFB_2	314.1	ESI^+	315.1	287.1	35
				259.1	40
AFG_1	328.1	ESI^+	329.1	243.1	36
				311.1	32
AFG_2	330.1	ESI^+	331.1	313.1	33
				245.1	40
T-2	466.2	ESI^+	489.2	245.2	36
				387.2	29
OTA	403.1	ESI^-	402.1	358.1	-28
				211.0	-38
ZEN	318.1	ESI^-	317.1	175.1	-38
				131.1	-32

The samples were detected by the established MRM method.

Table S3 Matrix effects of mycotoxins in real sample.

Mycotoxin	Arecae nut		Coix seed		Platycladi Seed		Spine Date Seed		Barley		Malt		Peanut		Corn	
	Without MMON	With MMON	Without MMON	With MMON	Without MMON	With MMON	Without MMON	With MMON	Without MMON	With MMON	Without MMON	With MMON	Without MMON	With MMON	Without MMON	With MMON
AFB_1	-35.96±1.86	-12.85±1.62	-18.44±2.79	-10.31±1.68	-20.00±4.60	-1.81±2.14	-15.86±3.54	-5.48±2.06	-15.62±1.51	-7.93±4.28	-22.58±0.79	-9.12±1.72	-17±2.6	-6.56±3.25	-17.48±1.47	-7.52±13.78
AFB_2	-41.99±1.98	-13.47±2.57	-21.94±3.10	-14.43±1.73	-24.57±4.07	5.24±3.37	-19.7±4.24	-3.75±10.45	-20.00±2.66	-0.09±4.61	-29±1.99	-9.26±1.91	-22.09±1.72	-9.44±10.02	-21.89±1.31	-1.60±11.35
AFG_1	-40.61±2.12	-9.37±2.55	-19.87±3.84	-9.93±2.24	-22.50±5.14	-2.70±2.69	-18.46±3.54	1.52±3.19	-18.07±2.46	-10.97±4.24	-27.38±2.52	-11.65±1.59	-21.09±2.89	-12.82±7.72	-19.37±1.69	-5.02±10.11
AFG_2	-47.39±1.60	-3.84±3.02	-24.51±2.12	-5.79±1.43	-27.25±4.74	-5.56±3.27	-23.73±3.51	-1.85±2.7	-21.44±1.3	-11.29±3.22	-33.14±0.37	-12.87±1.98	-23.2±2.55	-4.54±8.89	-31.59±2.21	-16.91±9.39
T-2	-9.88±9.60	0.69±1.45	0.15±11.19	-2.94±2.17	-3.04±12.57	-8.76±5.20	1.58±8.89	-5.73±6.35	1.31±9.95	-5.44±1.98	-4.60±3.59	-5.34±6.34	12.37±7.97	-1.46±2.21	2.32±8.63	-13.63±6.01
OTA	-0.24±5.31	-12.28±4.90	-3.73±4.97	-11.85±3.30	-6.51±5.91	3.66±2.86	3.25±6.78	1.94±10.03	-0.48±1.31	-4.17±2.13	-6.27±1.63	-13.98±4.30	1.33±2.35	-3.61±2.74	-1.91±0.89	-11.75±4.5
ZEN	-8.96±3.51	3.17±4.15	-4.32±3.78	3.57±4.35	-12.27±5.96	-8.37±3.67	-2.61±2.7	-5.23±9.01	-1.73±1.74	-10.01±3.56	-11.67±1.51	-1.06±1.18	0.06±3.87	-11.35±7.78	-2.00±1.48	-12.79±11.05

The sample spiking concentration is 50 μg/L.

Table S4. Comparison of the proposed method with other analytical methods.

Adsorbent	Method	Matrix	Analytes	Linear range (μg/L)	Recovery (%)	LOD (μg/L)	Equilib-rium time(s)	Reusa-bil-ity(cy-cle)	Ref.
Fe3O4@UiO-66-NH2@MON	MSPE-HPLC-FLD	Corn, Rice, Millet	AFB1 AFB2 AFG1 AFG2	1-500	87.3–101.8	0.15-0.87	600	7	[23]
HMON	SPE-HPLC	Corn, Soybean, Millet, Rice	AFB1 AFB2 AFG1 AFG2	0.1–100	85-98%	0.03-0.04	600	/	[39]
Fe3O4@MON-NH2-OH	MSPE-HPLC-DAD	Tradi-tional Chi-nese Medi-cine	ZEN	5–2500	80.7–106.0	1.4–35	420	/	[40]
PEG-MWCNTs-MNP	MSPE-UHPLC-Q- Exactive HRMS	Milk	13 myco-toxins	0.15–100	81.8–106.4	0.005–0.050	300	/	[41]
MHNTs@C=C@MIPs	MSPE-HPLC-FLD	Oat, Wheat	ZEN	10-200	75.0–88.4	2.5	600	/	[42]
AAOM	Dual-Mode Sensor	Beer	T-2	0.1-100000	97.1-108.7	0.056	1200	5	[43]
Au nanopop-corns/ Nafion-MWCNTs	Electrochemi-cal measure-ments	Malt	OTA	0.001–10	89.82-95.65	0.001	/	/	[44]
CPNM	Electrochemi-cal measure-ments	/	DON	0.01-10000	92.7-105.3	0.0096	300	/	[45]
Poly(DES)@MIL-101-NH_2(Cr)	MIP-SPE-HPLC-DAD	Coix seeds	ZEN	50-300,000	90.39-91.99	14.6	2160	7	[46]
MMON	MSPE-UPLC-MS/MS	Arecae nut, Coix seed, Platycladi Seed, Spine Date Seed, Barley, Malt, Peanut, Corn	AFB1 AFB2 AFG1 AFG2 T-2 ZEN OTA	0.01-100	81.32–116.10	0.002-0.15	10	10	This work

Note: anodic aluminum oxide membranes: AAOM; calcined PA-NH_2-MIL-101: CPNM.

Table S5 DFT-based analysis of solvation effects

Inter-Potential energy (kcal/mol)	Mycotoxin							
	AFB_1	AFB_2	AFG_1	AFG_2	T-2	DON	OTA	ZEN
In aqueous solution	-159.48	-148.26	-172.38	-146.54	-176.51	-119.11	-165.91	-123.09
In vacuum	-176.54	-160.39	-195.51	-147.28	-210.04	-168.27	-234.21	-153.01

Table S6 MMON synthesis cost and estimated yield per step

Step	Material	Amount per batch	Unit price (RMB)	Cost per batch (RMB)	Step cost (RMB)	Step yield (g, estimated)
Fe_3O_4 synthesis	$FeCl_3{\cdot}6H_2O$	2.7 g	90 RMB / 500 g	0.49	1.99	0.9–1.0
	$FeCl_2{\cdot}4H_2O$	1 g	10 RMB / 500 g	0.02	—	—
	NH_4OH	20 mL	370 RMB / 5 L	1.48	—	—
**Subtotal Fe_3O_4**	—	—	—	—	**1.99**	**0.9–1.0 g Fe_3O_4**
SiO_2 coating	TEOS	1 mL	1575 RMB / 2.5 L	0.58	—	—
	NH_4OH	5 mL	370 RMB / 5 L	0.37	—	—
	EtOH	320 mL	96 RMB / 5 L	4.81	—	—
**Subtotal $Fe_3O_4@SiO_2$**	—	—	—	—	**5.76**	**1.08–1.3 g $Fe_3O_4@SiO_2$**
MMON synthesis	$Fe_3O_4@SiO_2$	400 mg	—	—	—	—
	CuI	8.8 mg	1208 RMB / 1000 g	0.011	—	—
	$(PPh_3)_2PdCl_2$	33.6 mg	337 RMB / 250 mg	45.3	—	—
	TEPM	200 mg	6237 RMB / 5 g	249.5	—	—
	1,4-diiodobenzene	317 mg	772 RMB / 100 g	2.45	—	—
**Subtotal MMON**	—	—	—	—	**297.3**	**1.5–1.8 g per 3 batches**
**Per test (8 mg)**	**MMON**	**8 mg**	**—**	**—**	**—**	**≈ 0.197 RMB/test (~0.028 USD)**

Table S7 MSPE procedure time table

Step	Operation	Details	Time (s/min)
1	Adsorption	Mix 8 mg MMON with 5 mL sample/standard and vortex	10 s
2	Magnetic separation	Separate sorbent from supernatant	5 s
3	Discard supernatant	Remove liquid	10 s
4	First elution	Add 0.5 mL eluent + ultrasonication	4 min
5	Magnetic separation	Separate sorbent	5 s
6	Collect supernatant	Transfer eluent	10 s
7	Second elution	Repeat elution step	4 min
8	Magnetic separation	Separate sorbent	5 s
9	Collect supernatant	Transfer eluent	10 s
10	Nitrogen blow-dry	Evaporate collected eluent	5 min
11	Reconstitution	Redissolve in 0.2 mL for UPLC-MS/MS, filter (0.22 μm)	1 min
**Total estimated time per sample: ~14-15 min**			
